# Assessing changes in non-alcoholic sugary beverage prices in Agincourt following South Africa’s Health Promotion Levy: A pre- and post-implementation study

**DOI:** 10.1177/22799036251358299

**Published:** 2025-09-12

**Authors:** Chengetai Dare, David Canning, Micheal Kofi Boachie, Carlos Riumallo Herl, Susan Goldstein, Evelyn Thsehla, Karen Hofman

**Affiliations:** 1 SAMR/Wits Centre for Health Economics and Decision Science - PRICELESS SA, Faculty of Health Sciences, School of Public Health, University of the Witwatersrand, Johannesburg, South Africa; 2 Department of Global Health and Population, Harvard School of Public Health, Boston, MA, USA; 3 Erasmus School of Economics, Erasmus University Rotterdam, Zuid-Holland, The Netherlands

**Keywords:** health promotion levy, sugar-sweetened beverages, tax passthrough, non-communicable diseases, price changes, overweight, obesity, South Africa

## Abstract

**Background:**

Taxing sugar-sweetened beverages (SSBs) effectively reduces consumption when it leads to increased consumer prices and demand is sufficiently responsive, alongside other factors. Given the significant disparities in health outcomes between urban and rural provinces, this study to seeks to measure the changes in the prices of SSBs in rural South Africa and estimate the extent of the tax passthrough following the introduction of the Health Promotion Levy (HPL).

**Design and methods:**

We employed pre-post regression analyses techniques using the 2023 HAALSI Nutritional Establishment survey data.

**Results:**

The results show that the HPL led to an increase in prices (in real terms), and the price increase was more than that of the HPL. On average, the price of carbonated beverages increased by ZAR2.24 per litre (95% CI: 1.65–2.83) post the introduction of the HPL. However, the price increase was only registered during the period the HPL was introduced. The tax passthrough for carbonates was estimated at 1.87, implying that the tax was overshifted to consumers.

**Conclusions:**

This study shows that introduction of the HPL led to an increase in prices of carbonated beverages in Agincourt. The value of the HPL was however eroded over time by inflation. Considering that the effective tax burden of the HPL is substantially below the 20% minimum threshold recommended by the World Health Organisation, it is important that the government raises the HPL to maintain its value. Increasing the HPL is important to incentivise people to reduce SSB consumption.

## Introduction

The global intake of sugar-sweetened beverages (SSBs) has risen significantly in recent decades, with strong evidence connecting SSB consumption to the growing rates of overweight and obesity. These conditions are key risk factors for numerous non-communicable diseases (NCDs), including diabetes, high blood pressure, stroke, heart disease and certain cancers.^1–3^ By 2022, adult obesity had more than doubled since 1990 globally, whilst adolescent obesity quadrupled.^4^ Overall, 12.5% of the world population were obese in 2022.^4^ The greatest impact of obesity and its comorbidities is recorded in low- and middle-income countries.^5,6^

In South Africa, the rates of overweight and obesity are some of the highest within Sub-Saharan Africa. In 2016, 31% of adult males, 67% of adult females, and 13% of children under 5 years old were either overweight or obese.^7,8^ In the absence of any intervention, it is estimated that 50% of South Africans will be obese by 2030.^9^ In response to the growing rates of obesity and a variety of diet-related NCDs, in 2018, the government introduced a sugar-sweetened beverage tax, called the Health Promotion Levy (HPL) as a measure to reduce the intake of SSBs. The levy is applied to non-alcoholic sugary beverages (excluding fruit juices), at ZAR0.0221 per gram of sugar exceeding a limit of 4 g per 100 ml, constituting an effective tax burden of about 10% of the per-litre cost of the most widely consumed SSB at the time.^10^ The HPL is not adjusted annually to account for inflation to maintain the effective tax level. As such, the effective tax burden declined to approximately 8% of the per-litre price, as of June 2024.

SSB taxes are implemented in several jurisdictions including Chile, Denmark, Kenya, Mexico, Peru, the United Kingdom and several countries in Sub-Saharan Africa. Globally, as of July 2022, at least 108 countries apply taxes on at least one type of SSB.^11^ The taxes are expected to raise the retail price of SSBs, thereby reducing the affordability for the products, leading to a reduction in consumption. However, the success of these taxes hinges partly on how they affect the product’s retail price. Consumers adjust their buying habits based on changes in retail prices, not tax changes.^12^ The ability of tax policies to control SSB consumption depends in part on how taxes translate into higher prices (i.e. whether taxes adequately increase prices) and on the price elasticity of demand

Producers might react to tax policies in varied ways, based on their market influence and goals for maximising profits. They may raise the price by less than the tax increase, which is known as tax under-shifting). Producers may raise the price by the exact amount of the tax increase (exactly shifting or fully passed-through). Alternatively, taxes may be over-shifted (i.e. prices increase by more than the tax increase). The degree of passthrough thus determines the effectiveness of the excise tax in reducing SSB consumption (apart from the hypothetical cases in which demand or supply is perfectly price-inelastic).^12^ For example, a fully passed-through tax increase is more effective in reducing consumption than an under-shifted tax increase, while an over-shifted tax increase would be more effective than an equally-shifted tax.

Evidence from South Africa show that the HPL has caused significant price hikes for taxed SSBs.^1,13^ However, these findings are based on urban settings. As such, there is limited knowledge about SSB price changes in rural settings following the introduction of the HPL. We seek to fill this gap by measuring the changes in the prices of SSBs in Agincourt, a rural area in Mpumalanga Province, in South Africa. We also seek to estimate the extent of the tax passthrough following the introduction of the HPL. This particularly important considering the notable disparities in health outcomes between urban and rural regions.^14–16^ For instance, rural communities experience substantial obstacles in obtaining healthcare, such as financial constraints, limited transportation options, and long distances to medical facilities, compounded by a lack of available services.^14,15^ Many rural healthcare centres suffer from insufficient staffing and deteriorating infrastructure, which deepen existing disparities.^16^ Findings from this study may help policymakers review the HPL to improve health outcomes and reduce inequities between urban and rural communities.

## Methods

### Data

We used the longitudinal HAALSI Nutritional Establishment survey data^17^ collected from 1707 shops across 31 villages in Agincourt area, in Mpumalanga. The survey was conducted in three waves: 30 January 2017–10 March 2017, 08 March 2019−24 April 2019, and 29 October 2021−04 February 2022. The data contain information such as prices, beverage category and sugar content. We grouped the beverages into two (i.e. carbonates and bottled water), and stratified the data at village level for price variability. Overall, the dataset constitutes 190 observations (100 for carbonated drinks and 90 for bottled water). Table 1 depicts the summary statistics of the beverages across the villages.

**Table 1.** Summary statistics.

	Carbonates					Bottled water				
Village	*N*	Mean	Std. dev.	Min	Max	*N*	Mean	Std. dev.	Min	Max
Agincourt	4	10.95	0.49	10.29	11.41	4	10.19	1.62	8.20	12.17
Belfast	3	12.47	0.19	12.30	12.68	3	9.29	1.33	8.43	10.82
Croquetlawn	3	11.61	0.97	10.57	12.50	3	8.28	0.86	7.33	9.00
Cunningmore A	3	11.78	0.76	10.92	12.32	3	10.21	0.73	9.38	10.76
Cunningmore B	3	11.75	1.60	10.80	13.61	3	9.41	0.18	9.21	9.57
Dumphries A	3	9.71	1.20	8.72	11.05	3	10.16	0.87	9.17	10.78
Dumphries B	3	9.56	0.40	9.10	9.81	3	9.44	2.67	6.67	12.00
Dumphries C	3	10.33	0.35	9.95	10.62	2	9.33	3.77	6.67	12.00
Huntington	3	12.09	1.32	11.02	13.56	3	8.85	1.07	7.71	9.83
Ireagh A	3	11.91	1.20	11.09	13.29	3	9.07	0.61	8.44	9.67
Ireagh B	4	13.30	2.97	10.69	17.36	2	8.38	0.88	7.75	9.00
Ireagh C	3	11.29	0.96	10.21	12.03	2	9.33	3.77	6.67	12.00
Justicia A	3	12.75	0.98	11.91	13.83	3	8.93	0.31	8.62	9.25
Justicia B	3	12.30	1.26	11.30	13.71	3	10.73	1.25	9.50	12.00
Khaya Lami	3	11.01	2.02	8.90	12.92	3	9.65	0.98	8.89	10.75
Kildare A	3	11.73	0.94	10.65	12.36	3	8.33	0.08	8.24	8.41
Kildare B	3	11.87	1.51	10.84	13.60	3	9.44	0.64	8.79	10.06
Kildare C	3	12.39	1.07	11.50	13.58	3	9.90	1.07	8.67	10.58
Kumani	3	10.36	1.27	9.58	11.82	3	9.56	0.27	9.27	9.80
Lillydale A	4	11.80	1.64	10.40	14.13	3	9.89	0.75	9.03	10.45
Lillydale B	3	12.14	1.46	10.88	13.74	3	9.44	1.30	8.22	10.80
MP Stream	5	10.12	1.43	8.33	12.14	4	9.28	0.66	8.33	9.83
Makaringe	4	9.44	0.49	8.80	9.89	2	8.47	1.37	7.50	9.44
Newington B	3	10.87	0.81	10.09	11.71	3	8.81	1.12	7.78	10.00
Newington C	3	9.64	0.46	9.18	10.10	3	9.44	1.35	8.00	10.67
Rholane	3	11.23	1.26	9.79	12.10	3	9.89	1.33	9.06	11.43
Rolle C	3	10.86	0.47	10.36	11.28	3	9.92	0.20	9.70	10.07
Somerset A	4	12.75	0.82	12.25	13.97	4	9.88	1.06	9.00	11.42
Somerset B	3	10.96	0.90	9.96	11.71	2	8.17	0.24	8.00	8.33
Somerset C	3	10.83	1.89	9.60	13.00	2	12.00	2.83	10.00	14.00
Xanthia	3	10.06	0.91	9.53	11.12	3	8.57	0.41	8.33	9.04
Overall	100	11.28	1.49	8.33	17.36	90	9.45	1.29	6.67	14.00

### Sample size determination

This study uses secondary data, and as such there was no sample size calculation to determine the sample to be included in the study. The study sample is directly taken from the HAALSI Nutritional Establishment survey dataset.^17^

### Ethics approval for the study

In line with research ethics protocols, our study obtained an ethics waiver from the Human Research Ethics Committee at the University of Witwatersrand (HRECNMW24/08/04).

### Empirical estimation

We employ regression analyses to investigate SSB price changes following the introduction of the HPL. We adapt econometric models previously used by Stacey et al.^1^ and Clarke^13^ on similar studies. This entails two econometric models. The initial analysis is a straightforward before-and-after comparison aimed at identifying price changes after the HPL was implemented. The second model estimates the magnitude of tax passthroughs in relation to tax obligations for beverages subject to taxation.

### Price changes

To identify the changes in the prices of beverages after the HPL was introduced, we employed the regression model below:

(1) $$Price_{bvt}=\;\propto_{1}Post_{t}+\vartheta_{t}+Bev_{b}+\gamma_{v}+\varphi_{bvt}$$

where $Price_{bvt}$ is the average real price per litre for beverage category *b* in village *v*, in period *t*. The beverage category takes the value of 1 for carbonates, and zero for water. $Post_{t}$ is a binary variable indicating the implementation of the HPL intervention, assigned a value of zero for the period before HPL was introduced (1 April 2018), and one for the post-HPL period. $\vartheta_{t}$ represents the time trend of the period under study, while $Bev_{b}$ and $\gamma_{v}$ represents beverage category and village fixed effects, respectively. $\varphi_{bvt}$ is an error term.

As explained in Stacey et al.^1^ and Clarke,^13^ one possible risk to this regression model is the simultaneous one percentage point rise in the value-added tax (VAT) from 14% to 15%. This increase in VAT was applied to all the products, as such, it is unlikely to impact prices differentially across products. However, to account for any confounding effects to our estimates, the analysis in this study is conducted based on prices exclusive of VAT, calculated as follows. As in Stacey et al.,^1^ we assume that

(2) $$RetailPrice_{bvt}=Price_{bvt}*\left(1+VAT\right)$$

where $RetailPrice_{bvt}$ represents the selling price of beverage category *b*, in village *v*, period *t* and $Price_{bvt}$ represents the base price of the product, excluding VAT. The pre-VAT price is calculated by dividing the retail price by the VAT rate. Thus, the price measure is transformed as follows, to exclude VAT:

(3) $$Price_{bvt}=\{\begin{array}{c} \frac{RetailPrice_{bvt}}{1.14}\;if\;before\;April\;2018\\ \frac{RetailPrice_{bvt}}{1.15}if\;after\;April\;2018 \end{array}$$

The price measure is adjusted for inflation (2021 = 100) and container volumes, expressed in per litre terms.

### Tax passthrough

For a uniform specific tax, equation (1) would also provide the magnitude of the tax passthrough.^1^ However, the HPL is levied based on the level of sugar content, making it a variable tax. Thus, equation (1) only provides a measure of the extent of the price changes following the introduction of the HPL, and not the extent of the tax passthrough. To estimate the extent of the tax passthrough relative to the tax liability, we use the following regression model:

(4) $$Price_{bvt}=\;\propto_{1}Levy_{bt}+\vartheta_{t}+Bev_{b}+\gamma_{v}+\varphi_{bvt}$$

where $Levy_{bt}$ is the HPL rate per litre on beverage category *b* in period *t*. $Levy_{bt}$ is set to zero for the time before the HPL was implemented (1 April 2018). In other words, no tax liability applies to any products during that earlier period. After the HPL’s introduction. After the HPL’s introduction $Levy_{bt}$ is determined according to the sugar content, $SugarContent_{b}$ , as follows:

(5) $$Levy_{bt}=\{\begin{array}{c} 0\;\;if\;Sugar\;Content_{b}<\frac{4g}{100ml}\\ \left(Sugar\;Content_{b}-4\right)*0.0221*10\;if\;Sugar\;Content_{b}\geq \frac{4g}{100ml} \end{array}$$

From equation (4), the variable of interest is $\propto_{1}$ , which is a measure of the extent of the tax passthrough. A value of $\propto_{1}<1$ would imply that the tax is undershifted (i.e. the change in price is less than the change in the HPL). $\propto_{1}=1$ would imply a full passthrough. A value of $\propto_{1}>1$ would mean that the tax is overshifted.

We employed a fixed effects (FE) model to estimate the effect of the HPL on prices and the extent of the tax passthrough. To improve the robustness of the models, standard errors are clustered at village level. For sensitivity analysis, we ran two additional regressions: random effects (RE) and pooled Ordinary Least Square (OLS) models, and the results are largely similar.

## Results

As depicted in Table 1 an average price of ZAR11.28 (SD: ZAR1.49) for carbonated beverages, and ZAR9.45 (SD: ZAR1.29) for bottled water was estimated across the villages. Of all the villages, Ireagh B registered the highest price of carbonates (ZAR17.36) while MP Stream recorded the lowest price (ZAR8.33). The highest price of bottled water (ZAR14.00) was recorded in Somerset C while Dumphries B, Dumphries C and Ireagh C registered the lowest prices of ZAR6.67.

**Figure 1.**
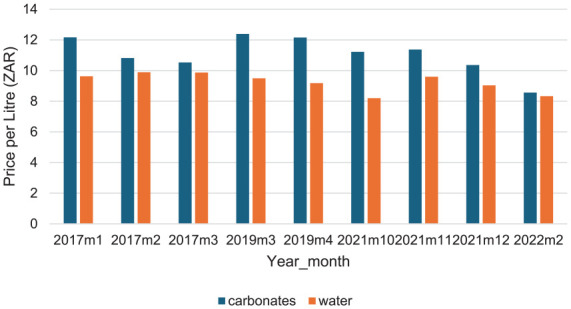
Nominal prices.

Figure 1 shows the nominal prices for the whole Agincourt area over the three wave-period. The graph shows a spike in the nominal price of carbonated beverages and a slight decrease in the price of bottled water in the period post 2017 – the period in which the HPL was implemented. The prices for both products declined steadily after 2019.

**Figure 2.**
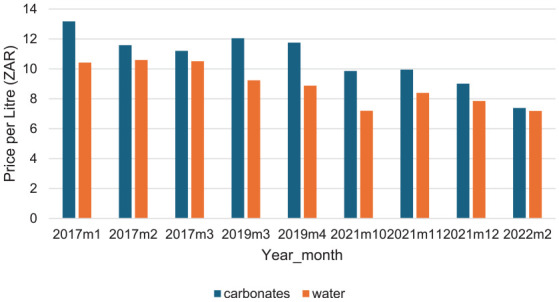
Pre-VAT prices.

Figure 2 shows the trend of pre-VAT real prices across the different waves. As in Figure 1, a significant increase in the price of carbonates was registered after 2017. While the price of bottled water has generally been decreasing over the wave periods, the price of decrease in the price of carbonates was recorded after post 2019.

The regression results on price changes following the introduction of the HPL (equation (1)) are depicted in Table 2, while Table 3 shows results on the extent of the tax passthrough (equation (2)). The results show that on average, the price of carbonated beverages increased by ZAR2.24 per litre (95% CI: 1.65–2.83) after the implementation of the HPL. On the other hand, the implementation of the HPL had no statistically significant impact on the prices of water. The coefficient of the time variable, $\vartheta_{t}$ , is negative and statistically significant for both carbonates and bottled water, implying that the price of both products has declined over time, which is in line with the trend shown in Figure 2.

**Table 2.** Regression results (the effect of the HPL on prices).

Variables	Carbonates			Bottled water		
	FE	RE	Pooled OLS	FE	RE	Pooled OLS
Post	2.242*** (0.290)	2.229*** (0.288)	2.191*** (0.291)	−0.443 (0.477)	−0.701 (0.470)	−0.701 (0.470)
Time	−0.079*** (0.006)	−0.080*** (0.006)	−0.081*** (0.006)	−0.034*** (0.009)	−0.031*** (0.010)	−0.031*** (0.010)
Constant	65.812*** (4.236)	66.395*** (4.207)	67.612*** (4.141)	33.938*** (6.271)	32.091*** (6.675)	32.091*** (6.675)
Observations	100	100	100	90	90	90
Number of villages	31	31		31	31	

Robust standard errors in parentheses.

*** *p* < 0.01. **p < 0.05. *p < 0.10.

**Table 3.** Regression results (tax passthrough).

Variables	Carbonates		
	FE	RE	Pooled OLS
Levy	1.873*** (0.243)	1.862*** (0.241)	1.831*** (0.243)
Time	−0.079*** (0.006)	−0.080*** (0.006)	−0.081*** (0.006)
Constant	65.812*** (4.236)	66.395*** (4.207)	67.612*** (4.141)
Observations	100	100	100
Number of villages	31	31	

Robust standard errors in parentheses.

*** *p* < 0.01. **p < 0.05. *p < 0.10.

Table 3 shows that a passthrough coefficient of 1.87 (95% CI: 1.38–2.37), implying that on average, a ZAR1 increase in the HPL was associated with a ZAR1.87 increase in the price of carbonates. As in Table 2, the time variable is negative and statistically significant, indicating a decreasing trend of the price of carbonates over time.

## Discussion

This study sought to measure the changes in the prices of carbonates and bottled water and estimate the extent of the tax passthrough following the legislation of the HPL in December 2017 and the introduction of the tax in April 2018. The results (Table 2) show that the introduction of the HPL was associated with a ZAR2.24 per litre (95% CI: 1.65–2.83) increase in the price of carbonated beverages. However, the price of bottled water has not been impacted by the HPL. These findings indicate that an HPL can be an effective policy strategy for increasing the prices of SSBs. The results are comparable to previous findings on the impact of the HPL on SSB prices,^1,13,18^ and similar studies on the impact of excise taxes on the prices of cigarettes^12^ and beer,^19^ which found that health taxes are effective in raising prices of the targeted products. However, our findings indicate the increase in the price of carbonates was experienced only for a short period (2018–2019).

Our findings indicate that the real prices of both carbonates and bottled water continued decreasing post the introduction of the HPL. The decrease in prices was evident in both nominal and real values. The price reduction could be a result of a marketing strategy by the industry/ retailers in light of the campaigns against SSB consumption, more especially in this rural setting. This is expected given that major players in the soft drink industry segment their market and charge different prices (price discrimination) to generate high revenue.^20^ The decrease was more in real prices (Figure 2) than in nominal prices (Figure 1) because of the eroding effect of inflation, since the HPL is not adjusted to account for inflation.

Importantly, the ultimate reductions in disease risk which health taxes are intended to achieve are determined by the extent market actors pass the tax through to the consumers. Our estimate of the tax passthrough for carbonates is 1.87 (or 187%), which shows that the tax is overshifted to consumers. This suggests that the producers of carbonates have control over the market. This is largely true considering that Coca-Cola Company controls approximately 60% of the market for carbonated beverages.^21^ Under such circumstances, the HPL can be an effective tool to reduce consumption of SSBs, particularly carbonates. Our results are consistent with the findings from Stacey et al.^1^ who found that the HPL was overshifted to consumers. However, Stacey et al. focused on urban consumers. Considering that the effective tax burden of the HPL is substantially below the 20% minimum threshold recommended by the World Health Organisation,^22^ it is important that the government raises the HPL and expanding it to fruit juices. To maintain its value, the HPL should be adjusted yearly for inflation. From a fiscal perspective, increasing the HPL and expanding it to fruit juices will help the government raise additional revenue for health expenditures and other fiscal requirements.

Although this study provides useful information on the potential effects of SSB tax policy measures, there are some limitations to consider. First, the dataset does not have a variety of untaxed beverages with which to compare the taxed drinks. Also compared to the few previous studies, our dataset is relatively small, with missing gaps over the period under study. The dataset is limited to carbonated drinks and bottled. It would have been more insightful if the dataset included other sugary drinks. Second, there could be other factors that influenced prices that were not controlled for in our analysis. However, we are not aware of any factor that could have influenced prices differently across these commodities. Further, our dataset is narrow and limited to Agincourt, making it difficult to generalise the findings to other rural areas in South Africa. Last, the fact that our study did not perform any sample size calculation to determine sample to be included in the analyses may bias the results. Despite these limitations, the findings are comparable to previous findings on the effects of the HPL in South Africa’s urban areas.

## Conclusion

With growing interest in the use of tax policy to raise the prices of SSBs and ultimately reduce the harms associated with SSB consumption, this study sought to measure the prices of carbonates following the introduction of the HPL. We found that the HPL led to an increase in prices (in real terms), and the increase was more than that of the HPL. However, the price increase was only registered during the introduction period of the HPL. Prices continued decreasing thereafter, largely due to inflation. To reduce the intake of SSBs, the government should consider raising the HPL from the current 8% of the retail price to the minimum 20% recommended by the World Health Organisation and expand the HPL to fruit juices. Increasing the HPL and expanding it to fruit juices are important in incentivising people to reduce the consumption of SSBs, while enabling the government to raise additional revenue for the fiscus.
